# Thermostable Lactonases Inhibit *Pseudomonas aeruginosa* Biofilm: Effect In Vitro and in *Drosophila melanogaster* Model of Chronic Infection

**DOI:** 10.3390/ijms242317028

**Published:** 2023-12-01

**Authors:** Elena Porzio, Davide Andrenacci, Giuseppe Manco

**Affiliations:** 1 Institute of Biochemistry and Cell Biology, National Research Council of Italy, Via P. Castellino 111, 80131 Naples, Italy; 2 CNR Institute of Molecular Genetics “Luigi-Luca Cavalli-Sforza” Unit of Bologna, 40136 Bologna, Italy

**Keywords:** lactonases, quorum quenching, *Pseudomonas aeruginosa*, biofilm inhibition, antimicrobial, *Drosophila melanogaster*, chronic infection

## Abstract

*Pseudomonas aeruginosa* is one of the six antimicrobial-resistant pathogens known as “ESKAPE” that represent a global threat to human health and are considered priority targets for the development of novel antimicrobials and alternative therapeutics. The virulence of *P. aeruginosa* is regulated by a four-chemicals communication system termed quorum sensing (QS), and one main class of QS signals is termed acylhomoserine lactones (acyl-HSLs), which includes 3-Oxo-dodecanoil homoserine lactone (3-Oxo-C12-HSL), which regulates the expression of genes implicated in virulence and biofilm formation. Lactonases, like Paraoxonase 2 (PON2) from humans and the phosphotriesterase-like lactonases (PLLs) from thermostable microorganisms, are able to hydrolyze acyl-HSLs. In this work, we explored in vitro and in an animal model the effect of some lactonases on the production of *Pseudomonas* virulence factors. This study presents a model of chronic infection in which bacteria were administered by feeding, and *Drosophila* adults were treated with enzymes and the antibiotic tobramycin, alone or in combination. In vitro, we observed significant effects of lactonases on biofilm formation as well as effects on bacterial motility and the expression of virulence factors. The treatment in vivo by feeding with the lactonase SacPox allowed us to significantly increase the biocidal effect of tobramycin in chronic infection.

## 1. Introduction

*Pseudomonas aeruginosa* is a Gram-negative, facultative aerobic rod-shaped bacterium, which belongs to the bacterial family Pseudomonadaceae and can be isolated from different environments, including soil, plants, and mammalian tissues [1,2]. *P. aeruginosa* is often described as “ubiquitous” or “a common soil and water bacterium” [3,4], but it is more often detected in environments affected by human activities [5]. It is a major cause of illness and death in humans suffering from immunosuppressive and chronic conditions. In these patients, *Pseudomonas* infections are difficult to treat due to the bacterium’s strong resistance to antibiotics and its propensity to form multicellular biofilm [6]. In fact, it is one of the top three causes of opportunistic human infections. A major factor in its prominence as a pathogen is its intrinsic resistance to antibiotics and disinfectants. *P. aeruginosa* has the largest bacterial genome sequenced so far, and its finely regulated metabolic systems reflect an evolutionary adaptation allowing it to thrive in different environments and resist a variety of antimicrobial substances [4]. *P. aeruginosa* healthcare-related infections include ventilator-associated pneumonia (VAP), intensive care unit infections, central-line-related blood stream infections, surgical site infections, urinary tract infections, burn wound infections, keratitis, and otitis media [7,8,9,10,11], and it is the main cause of mortality and morbidity in a debilitating genetic disease like cystic fibrosis [12]. *P. aeruginosa* is one of the six antimicrobial-resistant pathogens known as “ESKAPE” (*Enterococcus faecium*, *Staphylococcus aureus*, *Klebsiella pneumoniae*, *Acinetobacter baumannii*, *Pseudomonas aeruginosa*, and *Enterobacter* species) that represent a global threat to human health and are considered priority targets for the development of novel antimicrobials and alternative therapeutics [13]. The virulence of *P. aeruginosa*, like many other pathogenic microbes, is regulated by a chemical communication system termed quorum sensing (QS) [14]. QS is an interbacterial mode of communication accomplished through the coordinated production, secretion, and detection of chemical signals (QS signals or AutoInducers (AIs)) that trigger the expression of specific bacterial genes. One main class of QS signals self-produced by *P. aeruginosa* is termed acylhomoserine lactones (acyl-HSLs), which regulate the expression of genes implicated in virulence and biofilm formation [15]. *P. aeruginosa* harbors one of the most complex QS systems, equipped with at least four distinct but deeply intertwined and subordinated circuits [16,17] that control the activation of more than 300 genes in the *P. aeruginosa* genome [14]. The first two are N-acylhomoserine lactone (AHL) circuits, where Las and Rhl circuits, both activated by an increased cell density within a bacterial population, function in series to control the expression of virulence factors. The third, called the *Pseudomonas* quinolone signal (PQS) system, is triggered by iron limitation and interconnected with the previous two [11]. The three AI synthases, LasI, RhlI, and PqsABCDH, produce the AIs 3-Oxo-C12HSL, C4-HSL, and PQS, respectively. The AIs are detected by the cytoplasmic transcription factors LasR, RhlR, and PqsR, respectively. In particular, LasI produces a molecule, N-3-oxo-dodecanoyl-L-homoserine lactone (3-Ooxo-C12-HSL), which is detected by the cytoplasmic receptor LasR. RhlI produces N-butyryl-L-homoserine lactone (C4-HSL), which is recognized by the cytoplasmic receptor RhlR. The 3-Oxo-C12-HSL/LasR complex binds to the promoters of QS-regulated genes to control virulence factor production, including alkaline protease, elastases, toxin A, pyocyanin, and lasI itself. The Las circuit induces also the C4-HSL-controlled Rhl circuit, which in turn induces the expression of other factors, including rhamnolipid, pyocyanin, lectin A and B, elastases, exopolysaccharide, and RhlI itself [17]. Then, the fourth QS molecule, 2-(2-hydroxyphenyl)-thiasole-4-carbaldehyde, has been reported to be involved in the integrated QS (IQS) system, which in turn activates the expression of pqs genes. Although the synthesis of the IQS signal molecule and its bacterial receptor still remains elusive, this system can be triggered by phosphate starvation [15].

In general, *P. aeruginosa* adopts a planktonic lifestyle in acute infections or a sessile lifestyle during chronic infections. The latter is characterized by the formation of biofilm, very structured protection for bacterial cells, mainly composed of polysaccharides, lipids, proteins, and extracellular DNA (eDNA) [18]. Matrix components provide structural stability, facilitate liquid and nutrient transport, and support the robust integrity of a biofilm, including tolerance against antimicrobials, stress factors, and immune cells [19,20].

Several other mechanisms of virulence in *P. aeruginosa*, including motility (flagella and pili), immune evasion (elastase and alkaline protease), antibiotic resistance (pump efflux and modifying enzymes), cytotoxicity (hydrogen cyanide (HCN), exotoxin A, T3SS, and pyocyanin), iron scavenging (proteases and siderophores), and finally biofilm structure and dynamics (alginate and rhamnolipids), have been described [11].

Many of these mechanisms are regulated by QS mechanisms [21], and therefore antibiotic resistance and QS are possibly interconnected. In support of this hypothesis, some previous studies have highlighted potential synergistic effects between antibiotic treatments and interference in QS [22,23], still under investigation.

Numerous enzymes capable of hydrolyzing AHLs were isolated, characterized, and defined as quorum quenching (QQ) enzymes. Degradation usually occurs through the hydrolysis of the homoserine lactone ring by lactonases or through acylases that cleave the amide bond linking the homoserine lactone ring and the acyl side chain (Chen et al., 2013) [24].

Lactonase enzymes have been deeply characterized from an enzymatic and structural point of view [25,26,27,28].

The human Paraoxonases (PONs), particularly PON2, can efficiently hydrolyze 3 Oxo-C12-HSL [29]. As microbial enzymes, the phosphotriesterase-like lactonase (PLL) family, first discovered by some of us, includes well-characterized members able to hydrolyze AHLs, among them SsoPox [25,26]. Moreover, PON1 inhibits *P. aeruginosa* biofilm growth in an in vitro biofilm model [30] as well as in an animal model [31]. These observations indicate that lactonases, such as PONs, can protect the host from lethal *P. aeruginosa* infection. Here, we propose to exploit the quorum quenching ability of the phosphotriesterase-like lactonase SacPox from the thermophilic organism *Saccharolobus acidocaldarius*, already characterized for its lactonase activity [32]. We explored the effect on *Pseudomonas* virulence in vitro and in the animal model *Drosophila melanogaster*, which has been reported to be a good model for studying *P. aeruginosa* infections [33,34]. The reasons for this are as follows: (i) *D. melanogaster* displays evolutionary conservation of innate immune responses and NF-κB signaling cascades [35]; (ii) multiple genetic and molecular tools are available; it does not express endogenous lactonase enzyme or PON homologues [31]; and (iii) amenability to high-throughput screening, relatively low cost, and to date, no requirements for ethical approval.

In this study, we report the protective effect of the thermostable SsoPox and SacPox toward PAO1 infection, with respect to the control. Moreover, in the *Drosophila* chronic infection model, the addition by feeding of the purified SacPox reduces the biofilm formation in the crops of the infected flies. Finally, the treatment of SacPox together with tobramycin improves the effect of the antibiotic.

## 2. Results and Discussion

### 2.1. Expression and Purification of Phosphotriesterase-like Lactonases SacPox and SsoPox

The main aim of this work was to study comparatively the quorum quenching ability of the PLL SacPox, with respect to the first discovered thermostable PLL from our group, SsoPox, and the human lactonase PON2. Wild-type SacPox and SsoPox were purified to homogeneity with well-consolidated procedures, as reported in Section 3. In particular, starting from 8 L cultures of recombinant *E. coli* cells [32,36] through a combination of thermoprecipitation, separation by anion exchange chromatography, and exclusion molecular chromatography, about 65 and 42 total milligrams of SsoPox and SacPox were purified to homogeneity (more than 95%), respectively, as assessed by SDS-PAGE (Supporting Information Figure S1). Enzymes were used to evaluate the enzymatic activity on lactones and the effect on the virulence of *P. aeruginosa* PAO1.

### 2.2. Substrate Specificity on Lactones (TBBL, 3-Oxo-C12-HSL, C4-HSL)

During purification, enzymes were conveniently followed by a phosphotriesterase activity assay, one of the promiscuous activities of PLLs, according to a well-consolidated procedure [32]. We managed to determine the activity of these enzymes against the *Pseudomonas* quorum sensing molecules. An immobilized form of SsoPox wt has already been tested by another group [37]. Their results anticipated our idea that these enzymes are efficient quorum quenching enzymes. We therefore tested the free SacPox enzyme on the specific relevant HSL lactones, C4-HSL and 3-Oxo-C12-HSL. We made use of a titrimetric assay under a pH-stat mode. By measuring the pH reduction during the assay, at room temperature and pH 8.0, SacPox displayed a very low activity on C4-HSL, but it showed a high specificity constant (16.2 × 10^6^) on 3-Oxo-C12-HSL (Table 1). For comparison, data are reported also for SsoPox and recPON2.

### 2.3. Effect on Biofilm Formation

**Figure 1:**
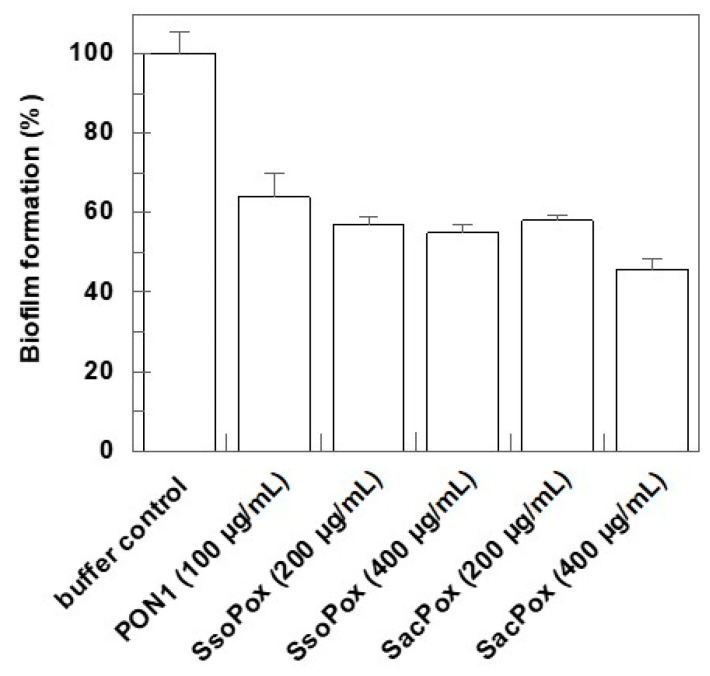
Biofilm inhibition assay on multiwell plate. Data are the means of three experiments within the indicated ranges (error bars).

To approach the in vitro study, a model for biofilm formation essentially adapting the procedure reported by Wang et al. [40] was set up. A crystal violet biofilm assay was used for biofilm quantitation. We tested the ability of thermostable lactonases to interfere with the biofilm formation of *P. aeruginosa* at the early stage of growth. In detail, an overnight PAO1 culture, in MHB medium, was refreshed in the morning and diluted to an OD_600nm_ of 0.2. Then, 200 µL of the culture was added to the wells of a 96-well plate. Different concentrations of SacPox and SsoPox (50–100–200–400 µg/mL) were tested. As a control, the same amount of the storage enzyme buffer (10 mM Hepes pH 8.5) was used. The experiments indicated that at 50 and 100 μg/mL there is no significant effect on biofilm formation, but at 200 µg/mL, both SsoPox and SacPox reduce the biofilm production by about 40% (Figure 1). As already reported by some of us, the human recombinant PON2 protein produced in *E. coli* after renaturation from inclusion bodies has a comparable effect already at 100 µg/mL, also in comparison with PON1 [39].

### 2.4. Effect of SacPox on Liquid PAO1 Culture: Growth and Pathogenicity Marker Analysis

A stab of *P. aeruginosa* PAO1 was grown in LB or MHB medium, and its identity was confirmed after growth on agar plates and by resistance to triclosan, as detailed in Section 3 [41,42].

Because we were interested in measuring the production of the pathogenicity markers pyocyanin and elastase, we preliminarily followed the growth of wild-type *P. aeruginosa* in the presence of different amounts of the two purified lactonases. Growth curves were followed at 37 °C. At this temperature and below, these thermostable enzymes, although less active, still maintain good activity. This allows comparison with the *Drosophila* temperature of growth (25 °C).

We added the purified enzymes directly into the *P. aeruginosa* growth medium, testing the effect on the bacterial growth. During the time of growth, portions of cultures were withdrawn in order to test residual phosphotriesterase activity (these experiments also gave us an idea of enzyme stability). The fate of specific markers of pathogenicity was followed in growing cells. In particular, we set up the conditions for pyocyanin and elastase extraction.

These experiments were performed only on SacPox because, as said above, a competitor group reported the same experiment for an SsoPox immobilized form [37]. The authors demonstrated a reduction in pyocyanin of 8–11-fold and elastase of 5-fold for the enzyme treatment with respect to the control, thus confirming the validity of our approach.

**Figure 2:**
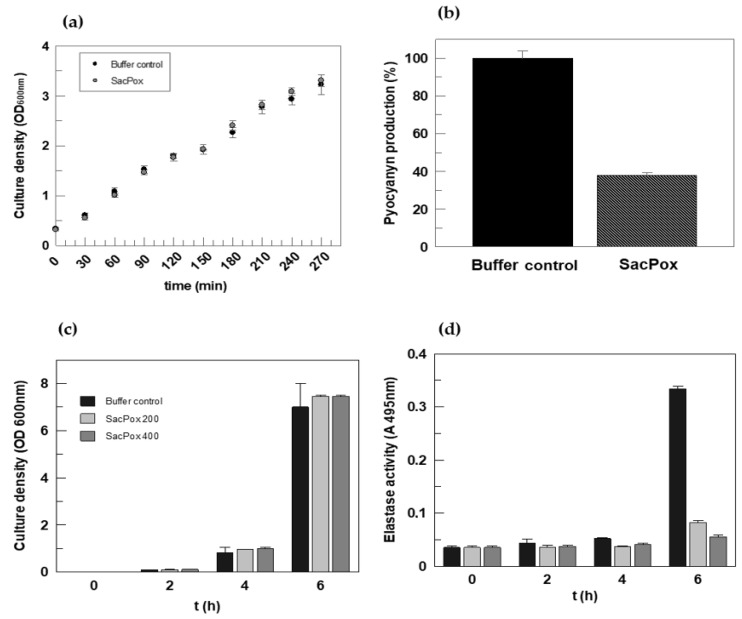
Effect of SacPox on the growth and virulence factor production of *P. aeruginosa* (PAO1). (**a**) Growth curve of PAO1 in LB medium, in absence or in presence of SacPox (140 μg/mL); (**b**) pyocyanin production (%) after 4 h of PAO1 liquid growth in PB medium in presence of SacPox (140 μg/mL); culture density (**c**) and elastase activity measurements (**d**) of liquid PAO1 growth in PB medium in absence and in presence of two SacPox concentrations (200 and 400 μg/mL). Data are means of three experiments within the indicated ranges (error bars).

Following the conditions outlined in Section 3, a colony of PAO1 was grown in LB medium, and after 16 h at 37 °C, the culture was diluted to 0.150 OD_600nm_, divided into two aliquots, one in the absence and one in the presence of the enzyme SacPox, and the OD was monitored every 30 min from 0 to 270 min. The enzyme added to the liquid culture (at a concentration of 140 μg/mL and more) did not affect the PAO1 growth, and it remained fully active throughout the incubation time (Figure 2a).

Pyocyanin is a metabolic by-product of *P. aeruginosa*, representing one of the more powerful factors of virulence; it is produced under the control of C4-HSL (which in turn is under the control of 3-Oxo-C12-HSL) [17]. The pyocyanin in the bacterial filtrates dissolves in chloroform, to which it confers a blue color [40].

The culture of PAO1, grown in PB medium for 16 h at 37 °C, was divided into two aliquots, one for the control and one for enzyme treatment, and at different timepoints, the pyocyanin was extracted following the procedure reported in Section 3. After 4 h of growth in the presence of SacPox (140 μg/mL), a 60% reduction in pyocyanin production was observed (Figure 2b); at the end of the experiment, SacPox maintained about 90% of its enzymatic activity, meaning that it was not inhibited nor inactivated by proteases.

The protease elastase encoded by lasB, secreted under the control of 3-Oxo-C12-HSL, plays an important role during *P. aeruginosa* infection, in particular in the initial phase of colonization of tissues with its capacity to degrade and inactivate components of the complement immune system [43].

For the determination of elastase activity, as described in detail in Section 3, the effect of SacPox (at 200 and 400 μg/mL) at 4 h and 6 h of growth was evaluated. The elastase production, which begins to be significant at 6 h of growth, undergoes a reduction of approximately 76% and 84% in the presence of 200 μg/mL and 400 μg/mL of SacPox enzyme, respectively (Figure 2c,d). After 6 h, the enzyme is stable and maintains over 90% of its specific activity (93 ± 2%); this result highlights the efficiency of the lactonase in reducing the virulence factor production, in particular in the first phase of *Pseudomonas* growth.

#### Evaluation of the Effect of SacPox on Swarming, Swimming, and Twitching Motilities

PAO1 moves on semi-solid surfaces following three types of motility: swarming, swimming, and twitching. The plates were prepared following the procedure reported in Section 3 and left to air dry for about 2 h before use.

**Figure 3:**
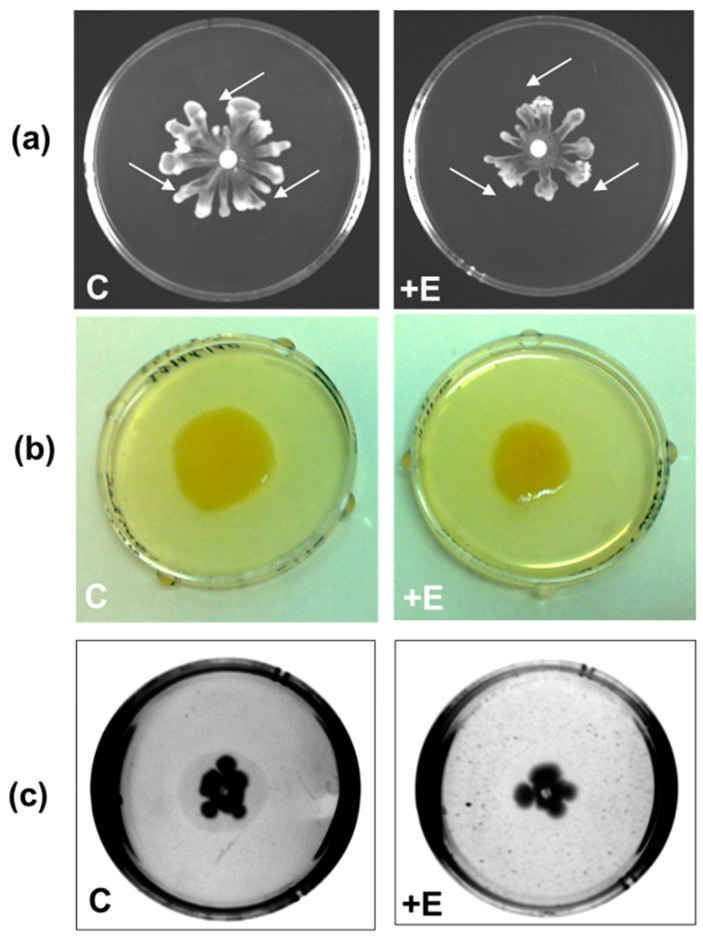
(**a**) Swarming plates. The arrows, added on the photos, indicate the points where the buffer (in “C”, control plate) and SacPox enzyme (in “+ E”, test plate) were was placed, drop by drop, on the surface of the NB/agar plate; (**b**) swimming motility and (**c**) twitching motility, both on LB agar plates in the presence of buffer control (in “C”, control plate) or SacPox enzyme (in “+ E”, test plate) added inside the growth medium.

For swarming motility, which is the surfing of the colony on the agar surface, PAO1 requires the movement of the flagellum; it also promotes bacterial adhesion to different surfaces, and consequently, the swarming motility is involved in the first phase of biofilm formation [40]. After being prepared, the plates were divided into “control” plates (“C” in Figure 3) and “test” plates (“+E” in Figure 3) where the enzyme was added. On the surface of the semi-solid agar plate, in the central position, 5 μL of a liquid PAO1 culture was placed, whereas at three points equidistant from the center, 6 μL of 10 mM Hepes pH 8.5 (for the control plate) or SacPox (for the test plate) was placed. After an overnight growth at 37 °C, the results obtained, as shown in Figure 3a, indicated that the presence of the SacPox enzyme on the surface reduced the swarming motility by about 35%.

Swimming motility is when *P. aeruginosa* penetrates into the agar from a preformed hole. In the “control” plate, the enzyme buffer was added before the medium solidified (10 mM Hepes pH 8.5), while in the “test” plate, the SacPox enzyme was added to the medium at a final concentration of 250 μg/mL, and it was able to reduce the swimming motility by about 20% (Figure 3b).

Under the same conditions as described above, the PAO1 twitching motility was also evaluated. The twitching motility, namely, the capability of PAO1 to infiltrate the space between the bottom of the agar and the plastic surface of the plate, depends on the type IV pilus [44]. As shown in Figure 3c, the interstitial zone, visible as a translucid halo, typical of twitching motility, is abolished in the presence of SacPox.

These results confirm the effect of SacPox on PAO1 motility, as a consequence of its quorum quenching ability.

### 2.5. PAO1-Induced Lethality in Wild-Type Flies Treated with Enzyme: Chronic Infection Model “by Feeding”

We set up the optimal conditions to create the right environment to allow the flies to be infected with PAO1 by putting 3 OD totals of PAO1 cells, resuspended in 100 μL of 5% sucrose and used to hydrate a paper disc put on the agar, at the bottom of the vials (see Section 3 for details).

**Figure 4:**
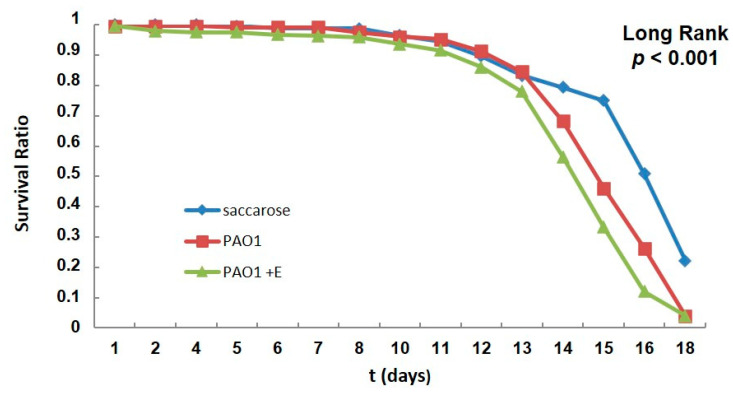
Survival curves post *P. aeruginosa* infection. Kaplan–Meier survival curves of adult *Drosophila* with PAO1 (red) and PAO1 +E (E: Enzyme SacPox) (green) with respect to the control (C: 5% sucrose) (blue). Experiments were performed with a minimum of 20 flies × 12 vials for each condition tested.

Flies were fed with PAO1 without or with the enzyme SacPox every 2 days, and fly survival was monitored over 18 days in response to the oral infection (Figure 4).

At 6 and 15 days post-infection, we sacrificed nine flies, which were resuspended in PBS, homogenized, and plated on PIA agar plates for CFU counting; we recorded a comparable number of CFUs in flies infected by only PAO1 and PAO1+ SacPox, corresponding to 956 and 912 CFUs/fly, respectively.

The treatment with SacPox does not kill *Pseudomonas* cells, but surprisingly, flies die more quickly compared to non-treated ones (Figure 4).

A possible explanation is that the enzyme reduces the biofilm formation, pushing the equilibrium towards more PAO1 cells in the planktonic state, which are able to spread faster in the body and kill the flies more quickly. This behavior is in line with the trend reported by Mulcahy at al. [19], where a non-biofilm-forming *P. aeruginosa* strain was significantly more virulent compared to PAO1, having a significantly increased rate of *Drosophila* killing, likely due to the virulence of the planktonic form [19].

#### Treatment with Enzyme SacPox and Antibiotic

In the chronic infection model “by feeding”, despite a comparable number of PAO1 cells recovered from the two groups of infected flies, treated or not treated with the enzyme, Pseudomonas in the presence of the enzyme was significantly more virulent compared to PAO1 alone, having a significantly increased rate of *Drosophila* killing.

The absence of biofilm protection could result in a higher susceptibility of cells to the antibiotic treatment. In the treatment of cystic fibrosis patients infected with *P. aeruginosa*, a formulation of aerosolized tobramycin has been reported to positively affect the reduction in bacterial growth [45,46].

Moreover, in a mice model of *P. aeruginosa* biofilm wound infection, tobramycin-loaded crystal nanoparticles demonstrated an enhanced clearance of *P. aeruginosa* from the wounds, which subsequently improved wound healing and the rates of reepithelization [47].

Tobramycin is an aminoglycoside antibiotic, effective against Gram-negative bacteria, especially the *Pseudomonas* species, but the minimal inhibition concentration (MIC) is affected by different factors such as the environment, the presence of other microorganisms, etc.

The biofilm mode of growth enables bacteria to evade the attacks from the immune system and to survive exposure to high concentrations of antimicrobial agents. It is commonly accepted that bacteria exhibit an up to 1000-fold increased tolerance towards a broad range of different classes of antibiotics under biofilm as opposed to planktonic growth conditions (conditional tolerance) (e.g., [48,49,50,51,52]).

It is also reported that sub-inhibitory concentrations of aminoglycoside antibiotics induce biofilm formation in *P. aeruginosa* and *E. coli* [53].

To test the effect of tobramycin in our animal model, we first tested the MIC in a liquid culture after overnight growth, which was 100 mg/mL (Supporting Information Figure S2) in our conditions.

**Figure 5:**
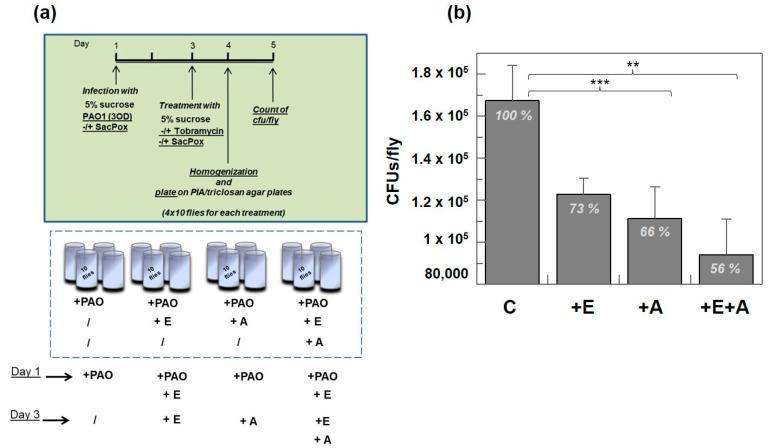
Treatment with enzyme SacPox and antibiotic tobramycin in the chronic infection model “by feeding”. (**a**) Scheme of treatment of the infected flies (4 × 10 flies, in triplicate for each condition) with only tobramycin (+A), only enzyme (+E), or both tobramycin and enzyme (+E+A); (**b**) number of PAO1 cells in the flies (CFUs/fly) after treatment in the three conditions (+E, +A, +E+A), with respect to the control (C). This analysis was performed in quadruplicate, in three independent experiments. *t*-test C/+A: 0.0008; *t*-test C/+E+A: 0.01. **: *p* < 0.01; ***: *p* < 0.001.

To evaluate if the presence of the lactonase SacPox could improve the effect of the antibiotic in an in vivo model by reducing the biofilm formation in the flies, we set up a treatment of *Drosophila* as in the scheme in Figure 5a. The enzyme was administered before the treatment with tobramycin (500 μg/mL) on day 1, and then, at 2 days post-infection, the groups “+E” and “+A” of infected flies were fed with only enzyme or only antibiotic, respectively, while the group “+E+A” was fed with both SacPox and tobramycin, with respect to a control group infected only with PAO1 on the first day.

After 4 days, 10 flies for each condition were sacrificed, homogenized in PBS buffer, and plated on PIA/triclosan agar plates to determine the number of CFUs/fly present after the different treatments. With three independent experiments of 40 flies for each treatment, in the presence of enzyme we had 73% of the CFUs/fly with respect to the control (Figure 5b); SacPox reduces the number of bacterial cells in flies, probably because by reducing the biofilm formation, the *Drosophila* immune response is more efficient and can manage the infection better. At a comparable level, the addition of tobramycin reduces the number of bacterial cells to 66% of the CFUs/fly with respect to the control; the treatment with SacPox and tobramycin contemporarily leads to a further decrease in the number of cells, corresponding to 56% of the CFUs/fly with respect to the control, suggesting a coordinated action of the enzyme and antibiotic, as expected (Figure 5b).

Our conclusion is that an early treatment with the enzyme allows for the management of the initial phase of the biofilm formation, reducing the protection of bacterial cells, which forced into the planktonic form are more susceptible to the action of tobramycin. Although we did not observe a complete additive effect of the enzyme and antibiotic, this first evidence confirms the ability of the treatment to control the infection by reducing in synergy the biofilm formation and the number of bacterial cells free to spread in the organism and diffuse the infection.

Unfortunately, it is known that bacteria are not entirely killed after antibiotic treatment but are still present as tolerant cells or “persisters” [54], and also, biofilm is a reservoir of such cells induced by different antibiotics. It was previously observed that *P. aeruginosa* which was isolated from a biofilm that was repeatedly exposed to ceftazidime exhibited the conventional form of resistance to antibiotics [55]. Therefore, the treatment with quorum quenching enzymes able to manage the biofilm formation is encouraging and represents a promising approach to both potentiate the effect of antibiotics and at the same time reduce the possibility that cells become “persister” cells.

## 3. Materials and Methods

### 3.1. Protein Expression and Purification

Large-scale expression and purification of the recombinant PLLs SsoPox and SacPox were carried starting from 8 L cultures of recombinant *E. coli* cells with well-consolidated procedures, as reported in detail in [32,36]. Soon after IPTG induction of protein expression, the cellular pellet was disrupted with a French press, and the supernatant after centrifugation was subjected to thermal precipitation steps and subsequent Q Sepharose Fast Flow and gel filtration chromatographic separation steps [32,36]. Purity was checked with SDS-PAGE. The 12.5% SDS-PAGE analysis was performed as described by Laemmli [56], at room temperature. About 65 and 42 tot mg of SsoPox and SacPox were obtained, respectively.

### 3.2. P. aeruginosa Growth and Biofilm Inhibition

A stab of *P. aeruginosa* PAO1 was grown in Luria–Bertani (LB) (Sigma-Aldrich, St. Louis, MO, USA) or Muller–Hinton Broth (MHB) (2 g/L beef extract, 17.5 g/L bacto caso-aminoacid, 1.5 g/L starch, CaCl_2_ 0.02 g/L, MgCl_2_ 0.01 g/L, pH 7. 3) at 37 °C in the absence and presence of 100 μg/mL ampicillin, and its identity was confirmed after growth on LB agar plates at 37 °C for 24 h and at 42 °C for 3h and by resistance to triclosan [41,42]. For biofilm quantification, PAO1 was grown in MHB for 18 h at 37 °C under rotary shaking at 225 rpm; then, the cell culture was diluted to 0.02 OD_600nm_ in fresh MHB, incubated at 37 °C under shaking up to 0.2 OD_600nm_, and aliquots of 200 μL were added to the wells of a 96-well microplate (Corning Inc., Corning, NY, USA). The purified enzymes SacPox, SsoPox, and PON1 (a kind gift obtained from our collaborator Florian Nachon from the Department de Toxicologie Institut de Recherche Biomédicale des Armées (La Tronche, France)) were added at different concentrations (100, 200, 400 μg), as indicated in Figure 1. For “buffer control”, the enzyme buffer (10 mM Hepes pH 8.5) was added in the same amount used for each enzyme. After incubation at 37 °C for 48 h, the liquid culture was removed from the wells by inversion, and the wells were washed three times with distilled water. The crystal violet staining was performed essentially as described by Nagant et al. [57], with some modifications. In the washed wells, the adherent cells were stained with a 0.1% crystal violet solution. After 15 min, the excess crystal violet was removed by inversion, and the wells were washed five times with 200 μL of sterile distilled water. The dye was dissolved with 200 μL of a 95% ethanol solution, and the absorbance of each well was read at 570 nm in a Victor3 PerkinElmer 1420 Multilabel counter (PerkinElmer, Waltham, MA, USA). Each condition was replicated in three different wells, and the experiment was carried out three times.

### 3.3. Lactonase Activity

Enzyme activity toward homoserine lactones C4-HSL and 3 oxo-C12-HSL was measured with pH titration using a pH-stat apparatus (T50 titrator model, Mettler Toledo, Columbus, OH, USA). The assays were performed at 25 °C, in 5 mL water adjusted to pH 8.5 with NaOH 0.1 M, containing 2% acetonitrile. Stock solutions of C4-HSL and 3-Oxo-C12-HSL were prepared by dissolving pure lactones in acetonitrile. Kinetic parameters were measured with substrate concentrations ranging from 0.1 to 1.2 mM; for each point, the blank was measured and subtracted. Assays were performed in duplicate or triplicate, and the results are the average of two independent experiments. The activity (Vmax) was defined as μeq min^−1^mg^−1^, which is the quantity of NaOH used to neutralize the free acids developed from the enzymatic reaction in one minute by 1 mg of SacPox. The SacPox lactonase activity on 5-thiobutyl-γ-butyrolactone (TBBL) was monitored in a 1 cm path length cell, using a reaction volume of 1 mL, in a Cary 5E spectrophotometer (Varian, Palo Alto, CA, USA), as reported in [25].

### 3.4. PAO1 Liquid Culture: Elastase and Pyocyanin Extraction

*P. aeruginosa* PAO1 culture was grown in LB medium at 37 °C under shaking for 16 h, then diluted at 0.2 OD_600nm_, divided into two aliquots, one in the absence and one in the presence of SacPox (140 μg/mL), and incubated at 37 °C under shaking. Every 30 min up to 4.5 h, bacterial growth was monitored by measuring the optical density at 600 nm. For the determination of the virulence factors, PAO1 was grown in PB medium (20 g/L bacto-peptone, 1.4 g/L MgCl_2_, 10 g/L K_2_SO_4_), diluted at 0.2 OD_600nm_, divided into two aliquots, one in the absence and one in the presence of SacPox (140 μg/mL), and incubated at 37 °C under shaking. In the absence of SacPox, the enzyme buffer was added as a control. After 4 h, for pyocyanin extraction, the protocol reported in [40] was used, with some modifications. In brief, 5 mL of culture supernatant was added to 3 mL of chloroform and mixed for 2 h at room temperature (RT). The sample was centrifuged at 8000× *g* for 30 min to separate the organic phase from the aqueous phase. The aqueous phase was discarded, and 1 mL of 0.2 M HCl was added to acidify the pyocyanin within the organic phase. This sample was mixed for 30 min at RT and was then centrifuged at 8000× *g* for 30 min: the upper acid phase was recovered, and the absorbance was read at 520 nm.

For evaluation of the elastase activity, an overnight PAO1 culture was diluted at 0.01 OD_600nm_ in fresh PB medium and divided into three aliquots for the three experimental conditions, buffer control (+10 mM Hepes pH 8.5), SacPox 200 (+SacPox at 200 μg/mL), and SacPox 400 (+SacPox at 400 μg/mL), and incubated at 37 °C under shaking, up to 6 h. Every 2 h, 2 mL of culture was collected, the bacterial growth was monitored by measuring the OD_600 nm_, and 1 mL of the culture was collected in microcentrifuge tubes and centrifuged at 15,000× *g* to sediment the bacterial cells.

The supernatant was filtered with a 0.22 μm filter, and 100 μL of this was used for the elastin Congo red (ECR) protease assays. One milliliter of the elastase reaction mixture was composed of 20 mg of elastin Congo red, 0.1 M Tris-HCl (pH 7.4), and 1 mM CaCl_2_. The mixtures were incubated at 37 °C for 4h under shaking. After the incubation, the samples were centrifuged at 15,000× *g* for 10 min to sediment the insoluble substrate. The absorbance of the supernatant was measured at 495 nm.

### 3.5. Motility Assays on Agar Plates

Swarming: Media used for the assay consisted of 8 g/L nutrient broth (3 g/L beef extract, 5 g/L peptone), 0.5% (wt/vol) Difco bacto-agar, to which 5 g/L of sterile glucose was added. Swarming plates were typically allowed to dry at room temperature for 2 h before being used. On the surface of the semi-solid agar plate, in the central position, 5 μL of a liquid MHB-PAO1 culture was placed, whereas at three points equidistant from the center, 6 μL of 10 mM Hepes pH 8.5 (for the control plate “C”) or of SacPox (16 mg/mL) (for the test plate “+E”) was placed. After overnight incubation at 37 °C, to evaluate the effect of the presence of the enzyme, the diameter of an imaginary circle created by the bacterium extended on the surface was measured in both the plates, and the percentage of reduction in the “+E” plate was calculated with respect to the control “C” plate.

Swimming: Medium used for the assay was LB that contained 0.3% (wt/vol) Difco bacto-agar. The plates were inoculated with bacteria from an overnight culture in LB agar (1.5%, wt/vol) plates at 37 °C with a sterile toothpick.

Twitching: Medium used for the assay was Difco LB broth (10 g/L NaCl, 10 g/L bacto triptone, 5 g/L yeast extract) with 1% (wt/vol) Difco granulated agar. The enzyme buffer (10 mM Hepes pH 8.5) and the enzyme SacPox (250 μg/mL) were added to the medium before solidification of the “control” and “test” plates, respectively. After preparation, the plates were briefly left to dry under a hood, and 2 μL of PAO1 overnight LB culture was inoculated into the bottom of the Petri dish, in a hole created by a sterile toothpick. After incubation at 37 °C for 18 h, the zone of motility at the agar–Petri dish interface was measured. In the white–black contrast image, the interstitial zone is more clearly visible in the “C” plate, and it is absent in the “test” plate.

### 3.6. D. melanogaster Infection Assays by Feeding

The wild-type *Drosophila* stock (Canton-S strain) used for the survival test was kept at 25 °C on a standard cornmeal/yeast medium. Infections were performed by using mid-log-phase LB cultures of *P. aeruginosa;* 3 OD totals of PAO1 cells were spun down and resuspended in 100 μL 5% sucrose and were spotted onto a sterile filter (Whatman) that was placed on the surface of 5 mL of solidified 3% agar in a plastic vial (VWR). The vials were allowed to dry at room temperature for approximately 30 min prior to the addition of *Drosophila*, as reported in [19]. Because of the high concentration of bacteria on the feeding discs and the possibility of bacteria forming aggregates on the feeding discs over time, male Canton-S flies (1–3 days old) were starved for 3 h prior to being added to vials (20 flies per vial). This ensured that *Drosophila* fed heavily on *P. aeruginosa* within the first couple of hours. It is therefore unlikely that the *P. aeruginosa* strains on the filters had sufficient time to form biofilms prior to being eaten by *Drosophila* and causing an infection. Male flies were fed with PAO1 (3 OD total, resuspended in 100 μL of 5% sucrose) without (“+PAO”) or with enzyme SacPox (400 μg/mL) (“+PAO+E”), with respect to the control fed only with 5% sucrose (“C”). For each condition, 240 flies (20 flies/vial, for 12 vials) were treated. Infection vials were stored at 25 °C in a humidity-controlled environment. Flies were relocated to fresh medium every two days, and dead adults were counted.

#### Treatment with Antibiotic Tobramycin

Flies were infected by feeding with 5% sucrose solution containing PAO1 (3 OD) at day 1 for all conditions, adding the enzyme SacPox (400 μg/mL) for the conditions “+PAO+E” and “+PAO+E+A”. After staying at 25 °C for 48 h, on day 3, we transferred the infected flies to new vials with 5% sucrose solution alone for “+PAO” or 5% sucrose solution containing the enzyme SacPox (400 μg/mL) for “+PAO+E” or tobramycin (500 μg/mL) (Sigma-Aldrich) for “+PAO+A” or both SacPox (400 μg/mL) and tobramycin (500 μg/mL) for “+PAO+E+A”. This experiment was performed in triplicate, treating 40 flies (4 vials with 10 flies each) for each condition. After 24 h, 10 flies for each condition were homogenized with a pestle in 200 mL PBS buffer; serial 10× dilutions of final fly extracts were cultured on PIA/triclosan agar, and colony forming units (CFUs) were counted after 24 h incubation at 37 °C. This analysis was performed in quadruplicate, in three independent experiments.

## Figures and Tables

**Table 1:** Activity of SacPox, SsoPox, and recPON2 on different lactones (at 25 °C, pH 8.0).

Substrate	Enzyme	Specific Activity (U/mg) [S] = (300 μM)	*k*_cat_ (s^−1^)	K_M_ (mM)	*k*_cat_/K_M_ (M^−1^ s^−1^)
TBBL 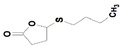	SacPox	58.3 ± 0.8	38.5 ± 0.74	0.024 ± 0.0005	1.86 ± 0.07 × 10^6^
	SsoPox	49.0 ± 11.2	29.0 ± 6.6	0.08 ± 0.003	3.6 ± 0.1 × 10^5^
	recPON2 ^b^	1.40 ± 0.07	1.10 ± 0.10	0.50 ± 0.15	2.20 ± 0.50 × 10^3^
4-undecanolide * 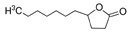	SacPox	1016 ± 33.3	912.8 ± 48.9	0.120 ± 0.010	7.6 ± 0.9 × 10^6^
3-Oxo-C12-HSL * 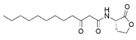	SacPox	2337 ± 9.5	2027.9 ± 5.5	0.120 ± 0.012	16.2 ± 2.2 × 10^6^
	SsoPox ^a^		1.01 ± 0.13	0.456 ± 0.128	2.2 ± 0.68 × 10^3^
	recPON2 ^b^	4.1 ± 0.4	2.7	0.50 ± 0.1	5.4 × 10^3^
C4-HSL * 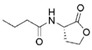	SacPox	n.d.	n.d.	n.d.	n.d.
	SsoPox ^a^	2.3 ± 0.2	n.d.	n.d.	11.62 ± 0.72
	recPON2 ^b^		1.53	0.6 ± 0.1	2.55 × 10^3^

* (pHSTAT); ^a^: from [38]. ^b^: from [39] (at 40 °C). n.d.: not detectable.

## Data Availability

Data are contained within the article and Supplementary Materials.

## Supplementary Materials

The supporting information can be downloaded at https://www.mdpi.com/article/10.3390/ijms242317028/s1.
