# Supplementary material for: Thermostable Lactonases Inhibit Pseudomonas aeruginosa Biofilm: Effect In Vitro and in Drosophila melanogaster Model of Chronic Infection
Source: Int J Mol Sci. 2023 Dec 1;24(23):17028. doi: 10.3390/ijms242317028 (PMC10707464; doi:10.3390/ijms242317028)
Supplement: Supplementary file 1 [file ijms-24-17028-s001.zip › ijms-2691743-supplementary.pdf]

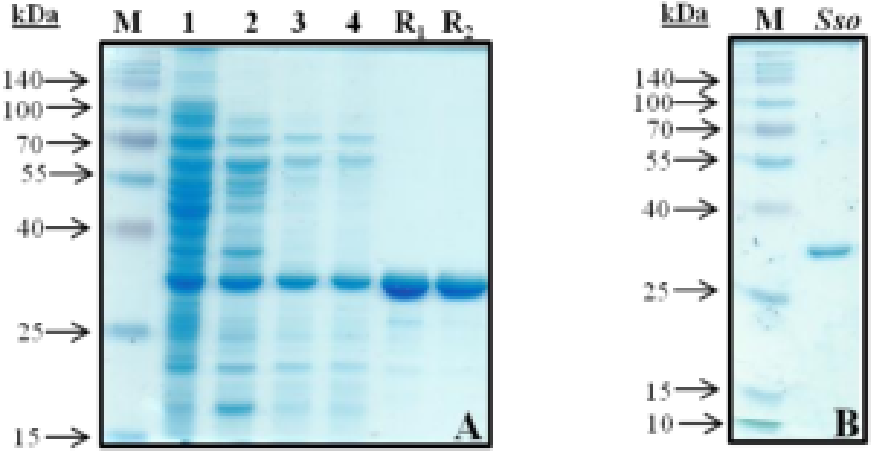

Figure S1: 12.5% SDS-PAGE analysis of protein fractions obtained during purification of SacPox (**A**) and SsoPox (**B**). M: molecular mass standards; 1: crude extract; 2: soluble fraction after thermoprecipitation at 60 °C; 3: soluble fraction after thermoprecipitation at 70 °C; 4: soluble fraction after thermoprecipitation at 80 °C; R_1: pooled Gf fractions: R_2: pure SacPox (5 μg); Sso: pure SsoPox (2.5 μg).

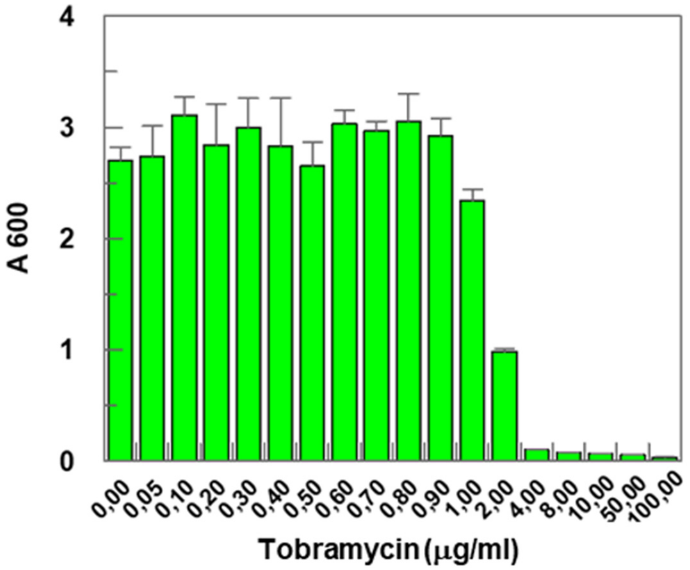

Figure S2: Bar graph of PAO1 overnight liquid growth in presence of different concentrations of tobramycin.
